# Poultry waste management practices in Bangladesh: Farmer’s perceptions, and food and environmental hazards

**DOI:** 10.5455/javar.2023.j654

**Published:** 2023-03-31

**Authors:** Jahan Ara Begum, Mohammed Nooruzzaman, Manasi Modak, Dolena Khanam, Ismail Hossain, Azmary Hasnat, Congreiv Kumar Kabiraj, Emdadul Haque Chowdhury

**Affiliations:** Department of Pathology, Faculty of Veterinary Science, Bangladesh Agricultural University, Mymensingh, Bangladesh; † These two authors contributed equally.

**Keywords:** Poultry, waste management, microbial contamination, hazards, farmers’ perceptions

## Abstract

**Objective:**

The poultry industry plays a key role in developing socio-economic and health sectors in Bangladesh. Poultry waste is a potential environmental threat as untreated poultry waste is used in vegetable gardens. The study aimed to investigate the current situation of small-scale poultry farms and their waste management practices in selected areas of Bangladesh and detect *Escherichia coli* and *Salmonella* in vegetables from farms using untreated poultry waste as fertilizer.

**Materials and Methods:**

A structured questionnaire-based survey was conducted in 86 small-scale poultry farms from different upazilas of Mymensingh and Khulna districts. 104 samples, including vegetables, poultry litter, water, and soil, were collected from vegetable gardens, ponds, fields, and wet markets in Mymensingh district to detect microbial contamination. Bacteria were identified based on their growth and colony morphology on selective media and motility tests. The presence of *E. coli* and *Salmonella* was confirmed by polymerase chain reaction (PCR) using a commercial PCR kit.

**Results:**

The survey revealed that mostly middle-aged males were involved in poultry farming. Most of the farmers had primary education and engaged in farming for about 5 years without training. In the study area, 37% of farmers collected droppings daily in the morning and used them as organic fertilizer. About 58% of farmers did not know the hygienic handlings of droppings and faced health problems. In PCR, either *E. coli* or *Salmonella* or both were confirmed in vegetables, litter, soil, and pond water.

**Conclusion:**

Appropriate poultry waste management practices can reduce the possible contamination of microbial agents in the human food chain.

## Introduction

The poultry industry is growing rapidly and establishing itself as a leading industry in Bangladesh [1]. The commercial poultry industry is a significant component of animal production that provides the nation with an affordable source of high-quality nutritious animal protein in terms of meat and eggs [2]. Despite significantly impacting our socioeconomic and health sectors, the poultry industry may also pose a potential threat to the environment [3,4]. To mitigate the costs of management practices, producers and researchers are investigating various strategies to address environmental sustainability and constraints while enhancing the value and portability of manure. Poultry manure is an excellent fertilizing resource as it contains high-quality nutrients, especially for supplementing nitrogen (N), phosphorus (P), and potassium (K). Manures decompose (mineralize) in the soil to ease nutrients for crop uptake. It is used to increase soil organic matter content that boosts the soil’s moisture-holding capacity, lowers soil bulk density, and improves overall soil structure, increasing the efficiency of crop production and irrigation [5].

In Bangladesh, gardens at home are an essential part of rural households. Farmers often use organic fertilizers made of cow and poultry manure directly in their vegetable gardens [6]. Poultry manure provides a great source of nitrogen, phosphorus, and trace elements [7]. It is a mixture of excreted manure with bedding materials, including sawdust, wood, wheat straw, and rice husks. Furthermore, poultry litter contains plant and animal nutrients, including nearly 30% crude protein, high levels of minerals, and some heavy metals [8,9]. Vegetables play a key role in ensuring food security. However, contamination of vegetables and crops with poultry litter or improperly composted poultry litter used in farms can be a source of pathogenic organisms such as *Salmonella* and *Escherichia coli* during the harvesting period that facilitates entry of pathogenic microorganisms into the food chain [10,11]. However, the most common agent linked to foodborne disease outbreaks is *Salmonella, *which poses a risk to human health and the environment [12]. These organisms colonize the gastrointestinal tracts of domestic and wild animals [13]. Although there is a continuous growth in the livestock industry in Bangladesh, poor waste management is an important constraint of the industry. Large amounts of poultry waste remain unutilized every day and become an obstacle in the poultry industry. Improper use of poultry waste is the most common source of environmental pollution and public health hazards due to a lack of awareness and proper disposal system [14]. The use of untreated poultry waste in vegetable gardens is the main source of contamination of vegetables. However, the original source is not clear yet. Therefore, this study was designed to investigate the current situation of poultry waste management in selected areas of Bangladesh and to detect the common microbial contamination of vegetables with *E. coli *and* Salmonella* spp. from farms using untreated poultry waste as fertilizer.

## Materials and Methods

### Ethical approval

The Animal Welfare and Experimental Ethical Committee of Bangladesh Agricultural University (BAU), Mymensingh, approved the experiment.

### Study design and area

A structured questionnaire-based survey was conducted in 86 small-scale commercial poultry farms from different upazilas of Mymensingh district such as Fulbaria (*n = *58), Gafargaon (*n = *24), and Terokhada upazila of Khulna district (*n = *4) during January to October 2020 to evaluate the recent status of poultry waste management practices. The study considered three categories of poultry farms, including broiler (*n = *37), layer (*n = *48), and cockerel (*n = *1). The data were collected from direct interviews with the respondents. The data included socioeconomic characteristics of farmers (age, gender, family size, level of education, main occupation, duration of farming, and training on poultry farm management), description of sampled farms (farm types, number of birds, quantity of litter, etc.) and environmental concern of poultry farming system (frequency of dropping collection, time for cleaning, cleaning responsibility, tools for cleaning, environmental effect, etc.). All the quantitative data were encoded in the Microsoft Excel program and then analyzed.

### Sample collection

A total of 104 samples including vegetable (*n = *72), poultry litter (*n = *17), pond water (*n = *5), and soil (*n = *10) were collected for detection of microbial contamination. The samples were collected from vegetable gardens near six poultry farms and one market in Mymensingh district during the period of January to October 2020. Farms where untreated poultry waste was directly used as fertilizer in their vegetable gardens and crop fields were selected. Samples were collected aseptically in sterilized containers and zipper poly bags, and transported immediately to the laboratory of the Department of Pathology, BAU.

### Isolation and identification of E. coli and Salmonella 

Isolation and identification of bacteria were carried out based on their growth pattern and colony morphology on selective and differential media, and motility tests. Transported samples were analyzed using morphological, cultural, and different biochemical tests following the standard protocol as described elsewhere [15]. Vegetable samples were cut into small pieces using sterile scissors, and soil and litter samples were collected using cotton swabs. Samples were inoculated in a screw-capped tube containing 5 ml of nutrient broth. Then the sample was incubated at 37°C overnight. After initial propagation and enrichment in nutrient broth, the samples were streaked on selective agar plates such as nutrient agar, *Salmonella-Shigella* (SS) agar, eosin methylene blue (EMB) agar, and blood agar. Petridishes were kept in the incubator at 37°C for 24 h. Agar plates were studied for the growth of microbes. On the surface of the agar, colonies were seen and a single colony of *E. coli* was further sub-cultured on sheep blood agar. The growth of *E. coli *and hemolytic reaction in the agar plates were examined after 18–24 and 48 h of incubation. The motility test was performed according to the method described by Cowan and Steel [16] to differentiate motile bacteria from non-motile ones. Briefly, one drop of cultured broth was placed on the coverslip and was placed inversely over the concave depression of the hanging drop slide to make hanging drop preparation. The hanging drop slide was then examined carefully under 100× using immersion oil. 

### Molecular identification of bacteria

Extraction of DNA from the colonies of bacteria was carried out as follows: two bacterial colonies were taken out of the plate and suspended in 5 ml of nutrient broth, and incubated overnight at 37°C. Then, 1 ml of the broth was taken into an Eppendorf tube and then centrifuged at 10,000 rpm for 5 min. After centrifugation, the supernatant was discarded and the pellet was suspended in 1 ml phosphate buffer solution and washed three times. The heating of bacterial suspension was performed at 100°C for 10 min following 10 min cooling step. Finally, the solution was centrifuged (at 10,000 rpm for 5 min) and the supernatant containing DNA was collected. DNA was stored at −20°C for further use.

For the polymerase chain reaction (PCR) of bacteria, a commercial PCR kit (Dream *Taq* PCR Master Mix, Thermo Scientific, USA) was used. The reactions were carried out in a total volume of 25 μl containing 12.5 μl of Dream *Taq* PCR master mix, 10 pmol/μl of each primer [17,18], 3.5 μl of nuclease free water, and 5 μl of DNA template. The initial denaturation step was carried out at 95°C for 5 min, followed by 35 amplification cycles of 95°C for 30 sec, 57°C for 30 sec, and 72°C for 1 min, with the final extension step of 10 min at 72°C. The PCR products were examined on 1.5% agarose gels prepared in 1 × Tris-acetate-EDTA buffer and visualized by ethidium bromide staining under ultraviolet light.

## Results

### Socioeconomic profiles of the sampled farmers

#### Age of the farmer

The farmers’ ages ranged from 21 to above 60 years. The age groups with the highest percentage (44%) of respondents were those between the ages of 41 and 50 years, while those with the lowest percentage (1%), were those beyond the age of 60.

#### Gender

In the socioeconomic and institutional spheres of developing nations, gender is a significant concern. Male owners were predominant (79%) than female owners (21%). Males are the owners of the poultry farms involved in managerial activities like immunization, medicine distribution, debeaking, and delivering chicks; while in most cases; women are more active in routine activities like cleaning cages, providing feed and water, and sorting eggs, etc.

#### Level of education

The education level in the survey area is grouped into six categories that including illiterate, primary, secondary, higher secondary, graduation, and post-graduation. Among the sample farmers, only 3% of respondents were illiterate. Approximately 52% of respondents had completed elementary school, and 30% had completed high school. About 11% of respondents had completed higher secondary education, and surprisingly, only 2% of them had earned a degree. According to the study’s findings, many young, educated people are now keen on starting a poultry farm. Education broadens people’s perspectives and inspires them to consider innovative approaches for better waste management.

#### Main occupation

The respondents were mostly involved in business and farming. In the research areas, only farming accounted for 51% of respondents, followed by business at 19%, farming and business at 11%, service (government) at 1%, and service (NGO) at 6%. Farming and other were 8%, while business and service (NGO) were 2%. It was observed that businessmen are fiscally sustainable enough to begin poultry farms.

#### Duration of farming

The duration of farming ranged from 1 to more than 10 years. Three categories of respondents were defined based on how long they had been farming. About 56% of farmers were involved in poultry farming for 1–5 years, 28% farmers were involved for 6–10 years, and 16% had been in this business for more than 10 years.

#### Management of poultry farming

In the study area, 35% of the respondents had received training for the management of their poultry farms, while 65% had not.

#### Description of the sampled farms

##### Farm types and number of birds

In this study, there were three distinct types of farms. These were broiler, layer, and cockerel. In the study area, layer farming was practiced by 55% of respondents, whereas broiler farming was practiced by 43% and cockerel farming by 2%. Four categories are used to illustrate the number of birds per farm (Table 1).

##### Dropping collection

The frequency of droppings collection depends on the flock size and type of birds. According to the study’s findings, broiler droppings were cleaned once a week or once a cycle, whereas layer bird droppings were cleaned every day or on an alternate day. The majority of the layer farms (37%) gathered droppings every day, whereas 27% collected every other day. Broiler farms, however, only collected droppings once per week (15%) and once a cycle (21%). Farmers observed that delay in dropping collection creates odor, nuisance, flies, etc.

##### Time and tools for cleaning

The majority of the respondents (71%) prefer to wipe the droppings in the early morning. Only 3% of respondents clean the droppings in the evening, compared to 26% of respondents who clean them around lunchtime. For droppings cleaning, the majority of the respondents use a shovel (*belcha*) due to its availability and affordable price. It was observed that 56% of respondents used only shovels for cleaning droppings while 23% used only water and 10% of respondents used a shovel and water for cleaning droppings. Only 1% of respondents used a brush, whereas 4% used both a shovel and a brush at the same time for cleaning. However, 8% of respondents said they used other tools like broom, duster, or mop for sweeping dust, dirt, and crumbs from the floor. 

**Table 1.** The number of birds per farm presented under the following four categories.

No. of birds	Broiler		Layer		Cockerel	
	Number	Percentage	Number	Percentage	Number	Percentage
500–1,000	4	11	2	4	0	0
1,001–2,000	16	43	11	24	1	100
2,001–3,000	7	19	15	32	0	0
Above 3,000	10	27	19	40	0	0
Total	37	100	47	100	1	100

##### Seasonal problems with cleaning

Most of the respondents (35%) faced the problem of dropping cleaning during the summer season. Whereas 33% of respondents found it problematic in winter and 26% in the rainy season. The common consensus among farmers is that during the summer, birds consume more water, which makes their droppings more watery and harder to clean. Due to damp weather in the rainy season, the droppings become wet and stick to the floor, posing a challenge throughout the cleaning process.

##### Place of waste disposal

Poultry wastes pose a threat to the environment that can be minimized through adequate management and utilization. In the study farms, about 9% of respondents disposed of their droppings on the drain side, while 28% disposed of on the roadside. On the other hand, 10% of farmers disposed of droppings in compost pits, while 13% disposed of composting in open spaces. A portion of the farmers (2%) disposed of the droppings directly to aquaculture pond while 11% farmers disposed of their poultry litter directly in agricultural lands. About 12% of the farmers in the sampled group used litter for biogas plants in addition to cooking fuel.

##### Use of droppings

Among farmers, 9.5% used droppings as fish feed, 9.5% used as both fish feed and as fertilizer in organic farms (Table 2). Droppings were used mostly as organic fertilizer in agricultural farms by 37% of farmers; however, 14% of farmers used them for biogas, fish feed, and cooking fuel. There were 11% of farmers sold the droppings while 19% of farmers gave litter free of cost. 

##### Inclusion of feathers into droppings

In 78% cases, dropped feathers were included in the droppings. The inclusion of feathers with droppings could be due to the problem of separation of feathers from droppings. 

##### Knowledge of farmers about hygienic aspect of poultry waste management

About 42% people had knowledge about hygienic handling of dropping, while 58% of farmers had no knowledge about it. Among respondents, only 48% of farmers used gloves during handling of droppings, while 52% did not use it.

### Health problems

Evidence exists that poultry manure or litter can carry a number of human infections, hence while managing poultry waste, biosecurity, and good hygiene have to be applied. In the study area, when farmers were asked about their health issues related to handling of poultry waste, farmers gave two different answers: 41% said they experienced disease outbreaks, and the remaining 59% said they never experienced any health issues related to waste handling. Few farmers experienced gas due to increased ammonia emissions, and some of them suffered physical deterioration and anorexia. Another group of farmers reported leg infections, discomfort in the eyes, and rashes. High ammonia emission from poultry houses leads to odor complaints from neighbors.

### Microbial contaminants in vegetables

For investigating the presence of microbial contaminants in vegetables, litter, soil, and pond water, we obtained 87 samples from six poultry farms that used untreated poultry manure in vegetable gardens, crop fields, and ponds. Another 17 samples were collected from common vegetable markets in the study areas. Isolation and identification of *Salmonella* spp. and *E. coli* in the samples were performed using standard procedure.

**Table 2.** Colony morphology of isolated *E. coli* and *Salmonella *spp*.* in different agar.

Media	*E. coli*	*Salmonella *spp.
Nutrient broth	Turbidity in the broth	Turbidity in the broth
Nutrient agar	Circular, smooth, colorless	Translucent, opaque, smooth colonies
SS agar	Slight pink smooth colonies	Opaque, translucent, colorless smooth round colonies with or without a black center
EMB agar	Yellow-green characteristic metallic sheen	Pink color colonies
Blood agar	Hemolysis	Complete hemolysis (β-hemolytic); partial hemolysis (α-hemolytic) and non-hemolysis (γ-hemolysis) of sheep RBC

### Isolation and identification of bacteria 

**Figure 1.**
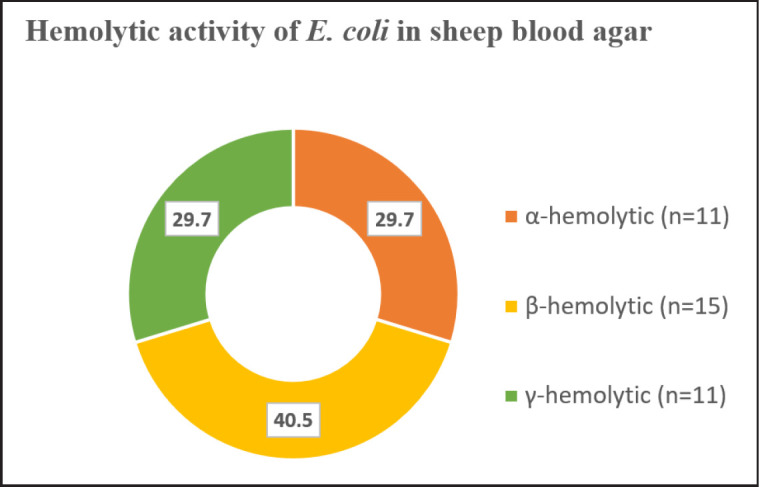
The hemolytic activity of the *E. coli* isolates on the sheep blood agar medium. The pie chart showing three different types of hemolysis produced by *E. coli* on blood agar medium including partial hemolysis (α-hemolysis), complete hemolysis (β-hemolysis), and non-hemolysis (γ-hemolysis).

**Figure 2.**
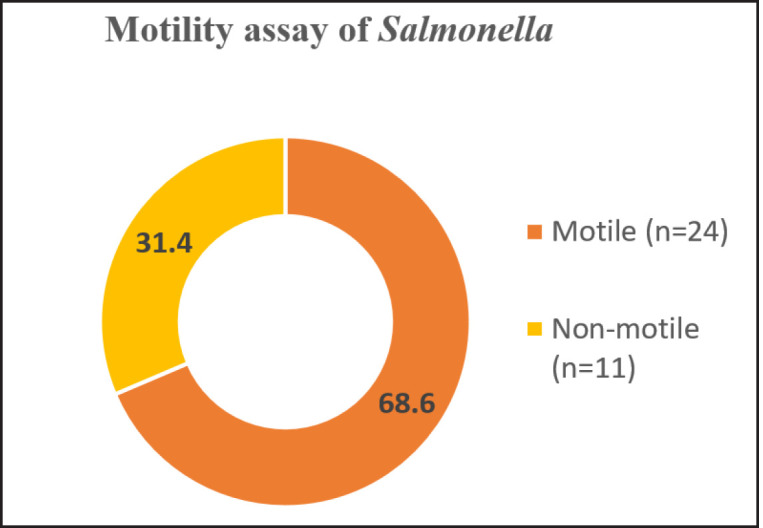
Graphical presentation of the percentage of motile and non-motile paratyphoid species under microscope. Motile *Salmonella *showed the characteristics of “jerking movement” under a microscope.

*Escherichia coli* and *Salmonella *were identified on the basis of the colony morphology in different agar plates (Table 2). The yellow-green characteristic metallic sheen in EMB agar and slight pink smooth colonies in SS agar were identified as *E. coli*. Hemolysis on blood agar is one of the many virulence factors of *E. coli *[19]. Therefore, the hemolytic activities of the selected *E. coli* isolates were tested on blood agar medium using sheep RBC where they were found to produce complete (β-hemolytic), partial (α-hemolytic), and non-hemolysis (γ-hemolysis) of sheep RBC in blood agar. Among them, most of the *E. coli* (40.5%) were β-hemolytic (Fig. 1), indicating enteropathogenic nature of the isolated *E. coli*. Furthermore, poultry *Salmonella* species under the group parathyroid are known to possess zoonotic significance and can contaminate human food chain [20]. Pink color colonies in EMB agar and opaque translucent colorless smooth round colonies with or without a black center in SS agar were characteristics of *Salmonella* species. The motility assay confirmed the presence of motile (68.6%) and non-motile (31.4%) *Salmonella *(Fig. 2). Motile *Salmonella *showed the characteristics of “jerking movement” under a microscope.

### Polymerase chain reaction

The presence of microbial contaminants in vegetables, litter, soil, and pond water was confirmed by PCR. The sampled farms used untreated poultry manure in their vegetable garden, crop field, and ponds. A total of 17 litter samples (11 untreated and 6 treated) were collected and screened for the presence of *E. coli* and *Salmonella*. Four of the untreated litters contain *E. coli* and 2 litters contain *Salmonella. *Interestingly, 6 litter samples collected from the compost pit showed no growth of tested bacteria. We also collected 10 soil samples from the vegetable gardens using untreated poultry waste as fertilizer. In accordance with litter samples, 2 soil samples showed growth of *Salmonella *(Table 3). Our preliminary survey showed that farmers were using untreated poultry waste in their ponds as feed additives for fish production (Data not shown). We collected pond water from respective farms and analyzed it for the growth of bacteria. The growth of *E. coli* was confirmed in one pond sample, and another one pond sample showed the growth of both *E. coli *and* Salmonella*. Then we collected 72 vegetables including Spinach (Palangshak), Green chili (Morich), Brinjal, Tomatoes, Coriander (Dhonia), Mustard leaf, Bitter gourd (Korola), Cabbage, Red spinach (Lalshak), Lemon, Okra (Dheros). Forty-four out of 72 vegetable samples showed the growth of either *E. coli *(*n = *15),* Salmonella *(*n = *15), or both* E. coli *and* Salmonella *(*n = *14) (Table 3). 

**Table 3.** Microbial contaminants in the litter, soil and pond water, and vegetable samples collected from farms using untreated poultry waste in their vegetable gardens.

Sample type		Number	*E. coli*	*Salmonella*	*E. coli* + *Salmonella*	Negative
Litter	Untreated	11	4	2	0	5
	Composted	6	0	0	0	6
Soil		10	1	2	1	6
Pond water		5	1	0	1	3
Vegetable		72	15	15	14	28
Total		104	21	19	16	48

## Discussion

A questionnaire-based survey was carried out in 86 small-scale commercial poultry farms in Mymensingh and Khulna districts from January to June 2020 to assess the state of poultry waste management in the study area. Our survey revealed that mostly middle-aged males (41–50 years) were involved in poultry farming. They mostly ran the poultry sheds, while women took care of the daily activities of poultry farms including feeding and cleaning. Although most of the farmers have primary education in the studied area, many young and educated people are now interested in poultry farming which leads the farmers to explore new ideas for better farm waste management that agreed with the results of Modak et al. [21]. About 42% of farmers were aware of the detrimental effects of poultry wastes on human and animal health as well as in the environment. Our result contrasts with a prior study that found that 95% of farmers are aware of waste management [14]. These disparities could be attributable to the geographical locations of the study area as well as the farmers’ educational backgrounds and farming experiences.

In our study, the majority of farmers collect droppings daily (37%) or on an alternative day (27%). The quality of nutrients in the litter is directly influenced by how often droppings are collected [22]. About 54% of farmers had some idea about hygiene and awareness of careful handling of poultry waste, although only 48% of farmers used gloves while 52% of farmers did not use any protective measures. About 41% of farmers faced health trouble while 59% never faced any health trouble. The majority of farmers disposed of their poultry droppings on the roadside (28%) and drain side (9%) directly, while 10% used that in the compost pits. Akter and Uddin [2] showed that 20% of farmers could not use their droppings for any particular work, 40% of them sold, 30% of them used for crop production, and 10% used for fish culture. Biogas production was done by 12.5% of farmers [23]. In Bangladesh, most of the small-scale commercial poultry farms manage their waste in an unorganized manner. The waste is either dumped in open spaces without any sort of treatment or used as fish feed or as fertilizer on nearby agricultural land and sold to the buyer [24]. However, the large poultry breeder companies in Bangladesh including Nourish Poultry and Hatchery Ltd., Aftab Bahumukhi Farms Ltd., Kazi Farms, Paragon Group, and others, have close connections to regional brokers who buy discarded litter and sell it to farmers of crops and fish [4]. Two-thirds (65%) of the respondents received no formal training in handling and management of poultry wastes. Besides, most of the smallholder poultry farmers started poultry farming without having prior training in poultry rearing which plays an important role in making poultry farming successful. However, a lack of knowledge of environmental and health aspects of poultry waste hindered successful waste management in study areas. 

In the study, tested vegetables showed the growth of either *E. coli*, *Salmonella, *or both *E. coli* and *Salmonella*, indicating severe public health hazards of the vegetables. Developing countries usually lack proper disposing guidelines for these wastages, which leads to the direct application of the litter to the agricultural fields and ponds. Furthermore, poultry waste is released directly to ponds and rivers through waterline, which contaminates the surface and drinking water with heavy metals, antibiotic residues, and microbes [25,26]. In addition, the majority of rural farmers are unaware of the potential hazards of untreated poultry litter. In Bangladesh, very few farmers used lime to treat droppings before disposal. Therefore, vegetables can be contaminated with pathogenic organisms at all stages of manufacturing and processing. Chen et al. [27] stated that there was a recognized potential risk for the on-farm transfer of pathogens to food. Infection of animals with zoonotic agents facilitates the excretion of pathogens through their feces that have been linked to several incidences of human foodborne illness. A recent British survey has shown that there was a one-in-three chance that a sample of livestock and poultry waste will contain either *Listeria*,* Campylobacter*,* Salmonella*,* Giardia*, or* E. coli* [23]. The microbiological risk associated with waste spreading should also be considered to determine the best way of litter material disposal. We collected litter, soil, water, and vegetables from the farms using untreated poultry litter as manure in their vegetable gardens or fields and ponds. It is noteworthy that, untreated litter contained *E. coli* and *Salmonella* while treated litter samples from the compost pit showed no growth of either *E. coli* or *Salmonella*, indicating the beneficial role of effective poultry waste management. However, *E. coli* and *Salmonella* did not characterize further. Effective poultry waste management techniques such as composting can assist to reduce environmental and health hazards by destroying most human and animal pathogens including *E. coli* and *Salmonella* [28–30]. Composting was shown to generate 160°F–170°F temperature and thereby kill most of the pathogens including *E. coli* and *Salmonella* [28–30]. Therefore, appropriate poultry litter management can reduce the possible contamination of zoonotic agents in the human food chain. However, the presence of *E. coli* and *Salmonella* in common vegetables from community market could be due to soiling of vegetables with contaminated water or unhygienic handling during harvesting and selling, or cross-contamination during pilling the vegetable in the shop.

## Conclusion

The present study described the profiles of small to medium-scale poultry farms and farmers, and their waste management practices in Bangladesh. Bacterial contamination like *E. coli *and *Salmonella *was detected in litter, vegetables, water, and soil near poultry farms where untreated poultry wastes were used as either fertilizer or disposal of droppings in ponds or lands. This served as a warning to the public health community to focus on the safe handling, transportation, and consumption of vegetables that are at risk of contamination.

## List of abbreviations

DNA, Deoxyribonucleic acid; EMB, Eosin Methylene Blue; SS,* Salmonella-Shigella*; NGO, Non Government organization; PCR, Polymerase chain reaction; RBC, Red blood cells.
